# Aerobic exercise attenuates insulin resistance via restoring branched chain amino acids homeostasis in obese mice

**DOI:** 10.3389/fnut.2024.1451429

**Published:** 2024-11-20

**Authors:** Wei Cao, Yajin Liu, Hao Wei, Yunfeng Dong, Haipeng Sun, Xuejiao Zhang, Junqiang Qiu

**Affiliations:** ^1^Department of Exercise Biochemistry, Exercise Science School, Beijing Sport University, Beijing, China; ^2^College of Sports and Health, Shandong Sport University, Rizhao, China; ^3^Chu Hsien-I Memorial Hospital, Tianjin Institute of Endocrinology, Tianjin Medical University, Tianjin, China; ^4^Center for Cardiovascular Diseases, The Province and Ministry Co-Sponsored Collaborative Innovation Center for Medical Epigenetics, Tianjin Medical University, Tianjin, China; ^5^Beijing Sports Nutrition Engineering Research Center, Beijing, China

**Keywords:** aerobic exercise, branched-chain amino acids, mammalian target of rapamycin, AMP-activated protein kinase, insulin resistance

## Abstract

**Introduction:**

Emerging evidences suggests that the disrupted branched-chain amino acids (BCAAs) homeostasis and elevated BCAAs promote obesity-related insulin resistance (IR). Exercise improves insulin sensitivity. However, whether BCAAs plays a role in the exercise-attenuated IR remains to be fully investigated.

**Methods:**

In this study, male C57BL/6J mice were induced to become diet-induced obese (DIO) and served as subjects. The initial investigation focused on the impact of exercise on IR and BCAAs. The DIO mice were randomly assigned to either a sedentary group (CON, *n* = 16) or an exercise group (EX, *n* = 16). The EX group underwent a 12-week aerobic exercise regimen on a treadmill. After 12-week, plasma BCAAs and branched-chain keto acids (BCKAs) were measured by liquid chromatography-mass spectrometry, glucose tolerance test (GTT) and insulin tolerance test (ITT) were performed, and the expression and phosphorylation of BCAAs catabolic proteins, as well as AKT T308 in gastrocnemius muscle and liver tissues, were evaluated using western blotting. Subsequently, the study explored the role of BCAAs in enhancing IR through exercise. Mice were randomly allocated into 4 groups: sedentary group (CON, *n* = 8), sedentary with BCAAs supplementation group (CON+BCAA, *n* = 8), exercise group (EX, *n* = 16), and exercise with BCAAs supplementation group (EX+BCAA, *n* = 16). The exercise protocol was as above. Mice in the BCAAs supplemented groups received drinking water containing 2% BCAAs. After 12-week, plasma BCAAs and BCKAs were measured, GTT and ITT tests were performed, and the phosphorylation of AKT T308, as well as p70S6K T389 in gastrocnemius muscle and liver, were compared between the EX group and the EX+BCAA group. Additionally, the phosphorylation of AMPKα T172 in both tissues was measured across all four groups.

**Results:**

12-week aerobic exercise improved insulin sensitivity in DIO mice while inducing BCAAs catabolic protein expression in skeletal muscle and liver, and reducing the plasma BCAAs level. Importantly, BCAAs supplementation elevated the plasma level of BCAAs and counteracted the exercise-attenuated IR. In skeletal muscle and liver tissues, BCAAs supplementation impaired the exercise-improved insulin signaling without enhancing mammalian target of rapamycin activity. AMPK activity was enhanced by aerobic exercise, which was abolished by BCAAs supplementation.

**Conclusion:**

Aerobic exercise attenuated insulin resistance via restoring BCAAs homeostasis and AMPK activity. The impacts of BCAAs intake on the metabolic effects of exercise sheds light on the combined exercise and nutrition intervention strategy for diabetes management.

## 1 Introduction

Metabolic diseases such as obesity, non-alcoholic fatty liver disease, and type 2 diabetes constitute a major public health challenge worldwide (1). Physical activity and exercise have been recognized as non-pharmacological interventions for metabolic disorders, including insulin resistance (IR) and diabetes (2–5). IR is a condition in which the body’s tissues and organs show a reduced biological action of insulin (6). IR is a major pathophysiological factor in the development and progression of Type 2 diabetes (7). Other than that, IR is strongly associated with numerus metabolic disorders (7).

Branched-Chain Amino Acids (BCAAs) refer to three essential amino acids: Leucine (Leu), Isoleucine (Ile), and Valine (Val). Traditionally, BCAAs have been widely used as supplements by athletes and sports enthusiasts (8). BCAAs contribute to protein synthesis by providing building blocks as well as stimulating mTOR pathway (8). BCAAs also improve exercise performance and alleviate exercise fatigue, partially by promoting glucose uptake and mitochondrial biogenesis (9).

In recent years, the suppressed BCAA catabolism and elevated BCAAs have been strongly linked with obesity, IR, and diabetes (10). The elevated BCAAs are a biomarker as well as a potential causal factor for the onset of IR and diabetes (11). BCAAs excessively activate mTOR, which suppresses the insulin signaling pathway (10). Enhancement of BCAAs degradation via pharmacological approach has been shown to attenuate IR in mice and humans (11, 12). Dietary intake of BCAAs also affects IR and diabetes in obese mice (13). These studies strongly suggest BCAAs metabolism is a therapeutic target for IR.

It has been reported that exercise affects BCAAs metabolism (14–16). The BCAAs catabolic pathway, consisting of greater than 40 enzymes in mitochondria, degrades excess BCAAs to maintain their homeostasis. Experiments show that exercise promotes the BCAAs catabolism in skeletal muscle and liver by promoting the activity of Branched-chain *α*-ketoacid dehydrogenase (BCKD), the rate-limiting enzymatic complex of BCAAs catabolic pathway (17–19). The effect of exercise on circulating BCAAs level remains controversial, which can be affected by the nutritional status of the subjects, exercise intensity, and exercise duration (15, 20–23).

In the current study, we showed that 12-week aerobic exercise reduced circulating BCAAs level in obese mice while attenuating insulin resistance. Aerobic exercise induced the expression of BCAAs catabolic protein in the skeletal muscle and liver. More importantly, supplementation of BCAAs in drinking water counteracted the effects of exercise on BCAAs and insulin sensitivity. These data demonstrated that aerobic exercise attenuated IR via restoring BCAAs homeostasis, and BCAAs or protein nutritional intake could tune the metabolic benefits of exercise.

## 2 Materials and methods

### 2.1 Animals

All experiments were performed in compliance with the protocol approved by the Internal Review Board of Beijing Sport University. 5-week-old male wild-type C57BL/6J mice were purchased from Huafukang Biotechnology Co., Ltd. (China). All mice were housed in a 22–24°C, 40–60% humidity and 12 h light/12 h dark environment with free access to water and standard chow. After 1 week of normal chow feeding for acclimatization, mice were fed a high-fat diet (Research Diets, USA) with a ratio of 60% fat for 12-week to induce obesity (DIO).

### 2.2 Study design

For the first experiment, DIO mice were randomly divided into sedentary group (CON, *n* = 16) and exercise group (EX, *n* = 16). The EX group undertook a 12-week aerobic exercise on a treadmill. Plasma BCAAs and BCKAs were measured, and glucose tolerance test (GTT) and insulin tolerance test (ITT) were performed after 12-week. At 48-h after the last exercise, subgroups from each group (*n* = 8) were administered an injection of either insulin solution or saline, and gastrocnemius muscle and liver tissues were sampled after 10-min of rest for further analysis.

**Figure 1:**
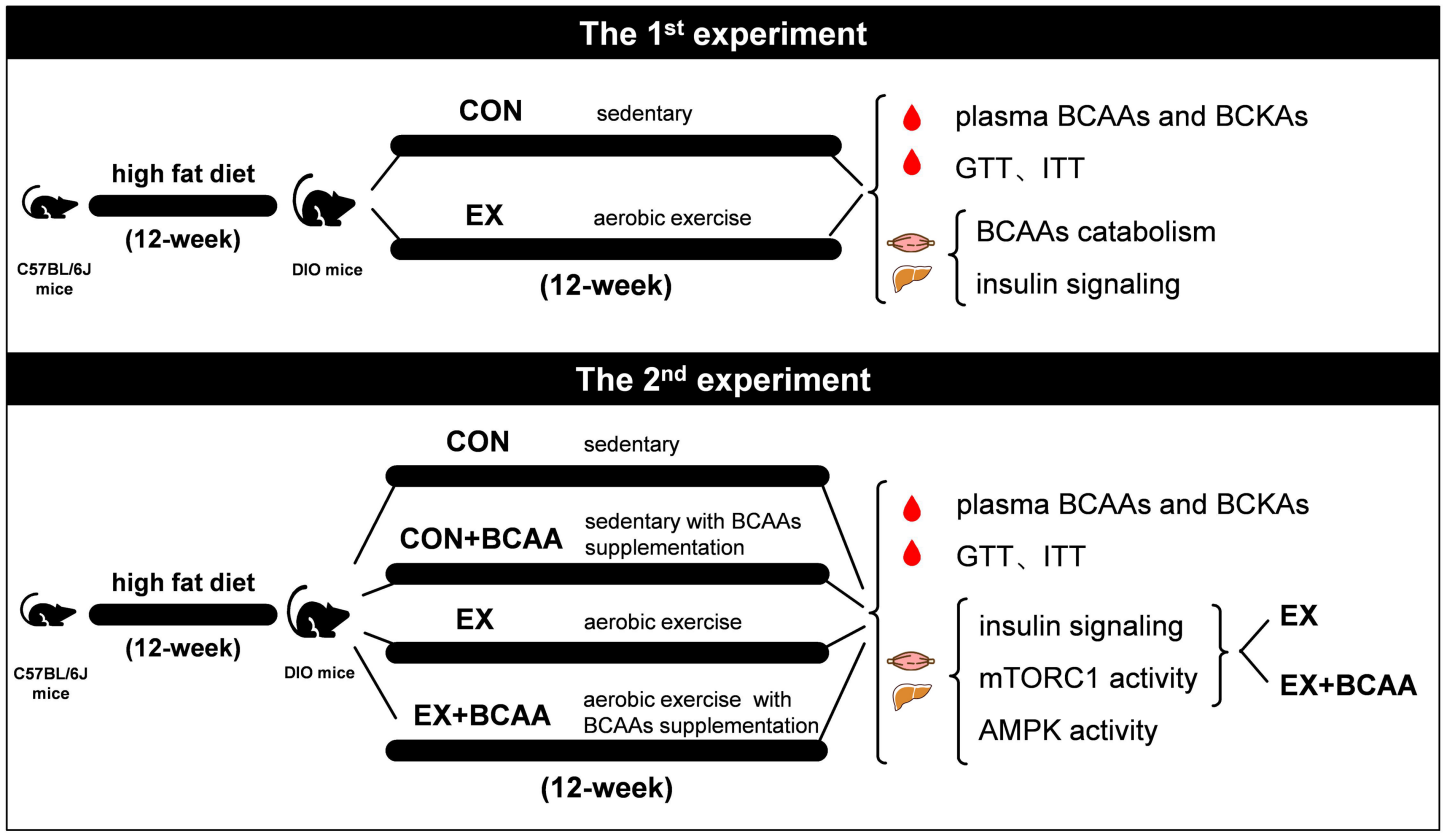
Experimental testing overview.

In the second experiment, DIO mice were randomly allocated into 4 groups: sedentary group (CON, *n* = 8), sedentary with BCAAs supplementation group (CON+BCAA, *n* = 8), exercise group (EX, *n* = 16), and exercise with BCAAs supplementation group (EX+BCAA, *n* = 16). Plasma BCAAs and BCKAs were measured, and GTT and ITT tests were performed after 12-week. At 48-h after the last exercise, subgroups (*n* = 8) from EX and EX+BCAA groups were administered an injection of either insulin solution or saline, respectively, and gastrocnemius muscle and liver tissues were sampled after 10-min of rest for further analysis (Figure 1).

### 2.3 Experimental protocol

#### 2.3.1 Exercise protocol

DIO mice were treated with sedentary or treadmill running aerobic exercise. DIO mice assigned to exercise were first run on a treadmill for 3-day to acclimate (10 m/min, 15-min), and then underwent 12-week treadmill running aerobic exercise training. The procedure consisted of 12 m/min for 40-min, 5-day per week. The training was performed between 15:00 and 17:00 in a day.

#### 2.3.2 BCAAs supplementation

For BCAA supplementation, DIO mice were supplied with drinking water containing 2% BCAAs (20 g/L, a 2:1:1 weight ratio of Leu: Val: Ile) (24). BCAAs were purchased from Sigma Aldrich (USA) with the following purities: “≥99.0%” for Leu, and “98.5–101.0%” for both Val and Ile. All water was replaced with fresh solution once weekly.

### 2.4 Body composition analysis

Body composition (fat and lean body mass) was analyzed using a Bruker Minispec mq 7.5 nuclear magnetic resonance analyzer (Germany).

### 2.5 GTT and ITT

Mice were deprived of food for 6 h. Blood was collected from the tail. A blood glucose meter (Roche, Germany) was used to measure the baseline blood glucose concentration. Mice were then intraperitoneally injected with 1 g/kg body weight of glucose (Sigma, USA) for glucose tolerance test (GTT) and 1.5 units/kg body weight of insulin (Novo Nordisk, Denmark) for insulin tolerance test (ITT). Blood glucose concentration was measured at 15-min, 30-min, 60-min, 90-min and 120-min after injection.

### 2.6 Blood and tissue collection

Blood was centrifuged at 5000 rpm for 10-min at 4°C to obtain plasma. Mice were sacrificed by cervical dislocation after blood sampling. The gastrocnemius and liver samples were quickly collected, and frozen in liquid nitrogen. All samples were stored at −80°C for future analysis.

### 2.7 Samples analysis

#### 2.7.1 Plasma treatment

Plasma BCAAs and BCKAs. All experiments were carried on a Shimadzu LC-20AD liquid chromatography (LC) system coupled to an API 3200 electrospray-ionization triple-quadrupole mass spectrometer (AB SCIEX, USA). The plasma levels of BCAAs and branched-chain keto acids (BCKAs) were detected by multiple reaction monitoring (MRM) in positive and negative electrospray ionization mode, respectively. Chromatographic separation was achieved on a ZORBAX SB-C18 (150 × 3 mm, 5 μm) column (Agilent, USA), and temperature controlled at 50°C. The standards and samples were separated using a mobile phase consisting of methanol/water (20:80, v/v) with 0.1% formic acid (eluent A) and acetonitrile (eluent B). An aliquot of 10 μL plasma was spiked with 110 μL methanol/acetonitrile/water (50:50:10, v/v/v) containing stable-isotope-labeled internal standards (48 ng [D3] Leucine, 24 ng [13C4, D3] KIV sodium salt, and 16 ng [D3] KIC sodium salt) and remained on ice for 10 min before being centrifuged at 14000 *g* at 4°C for 10 min. The supernatant was collected and dried with a stream of nitrogen. Following this, the samples were reconstituted in 100 μL of methanol/water (20:80, v/v) for analysis. The injection volume was 10 μL. Data acquisition and quantitation were performed with Analyst 1.7 and MultiQuant 3.0 software (SCIEX, USA), respectively.

#### 2.7.2 Western blotting

SDS lysis was used to extracted protein sample from gastrocnemius (skeletal muscle) and liver tissues. For western blot, protein samples were boiled and separated on 8–12% SDS-PAGE gels. After blocking with TBST containing 5% BSA for 2-h, the membranes were incubated with specific primary antibodies overnight at 4°C: BCAA transaminase 2 [BCAT2, 1:1000 dilution, Cell Signaling Technology (CST)], BCKDK (1:500 dilution, Santa Cruz Biotechnology), mitochondrial protein phosphatase 2C (PP2Cm, 1:1000 dilution, CST), BCKDE1α (1:1000 dilution, Santa Cruz Biotechnology), BCKDE1α S293 (1:4000 dilution, Abcam), protein kinase B (AKT, 1:1000 dilution, CST), AKT T308 (1:1000 dilution, CST), p70S6K (1:1000 dilution, CST), p70S6K T389 (1:1000 dilution, CST), AMPKα (1:1000 dilution, CST), AMPKα T172 (1:1000 dilution, CST), GAPDH (1:3000 dilution, Abways), *β*-tubulin (1:1000 dilution, CST). The membranes were then incubated with the following secondary antibodies for 2-h at room temperature: anti-mouse, anti-rabbit, and anti-goat (1:5000 dilution, Santa Cruz Biotechnology). Antibody-bound proteins were detected by chemiluminescence (Tanon, China). ImageJ Software was used to measure protein relative abundance.

### 2.8 Statistics analysis

Data were analyzed using SPSS 25.0 statistical software and presented as means and standard error. An unpaired Student’s t-test was used for comparison between the two groups. Two-way ANOVA followed by Bonferroni’s *post hoc* tests was used for the comparison between multiple groups. *p* < 0.05 was considered statistically significant.

## 3 Results

### 3.1 Aerobic exercise reduced plasma BCAAs level and increased BCAAs catabolic protein expression in DIO mice

**Figure 2:**
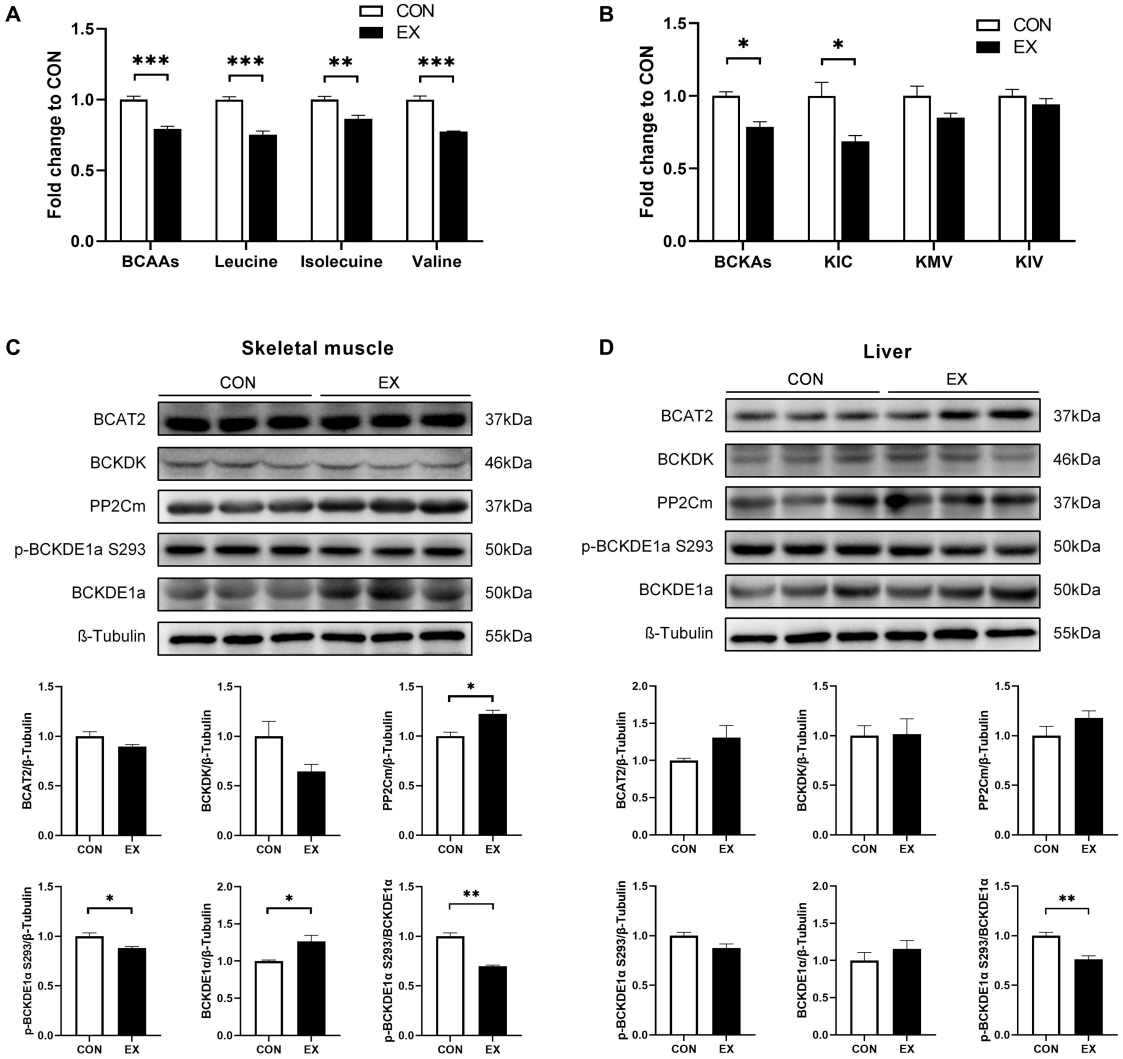
Effect of 12-week aerobic exercise on plasma BCAAs **(A)** and BCKAs **(B)** levels, and BCAAs catabolic protein expression in skeletal muscle **(C)** and liver **(D)**. *p < 0.05, **p < 0.01, ***p < 0.001. BCKA, branched-chain keto acids; KIC, α-ketoisocaproic acid; KMV, α-keto-*β*-methylvaleric acid; KIV, α-ketoisovaleric acid.

12-week aerobic exercise significantly reduced the plasma levels of BCAAs (Figure 2A) and their catabolites BCKAs (Figure 2B) in DIO mice. Meanwhile, exercise reduced the phosphorylation of BCKDE1α in both skeletal muscle (Figure 2C) and liver (Figure 2D), indicating increased BCKD activity.

### 3.2 Aerobic exercise enhanced insulin sensitivity in DIO mice

**Figure 3:**
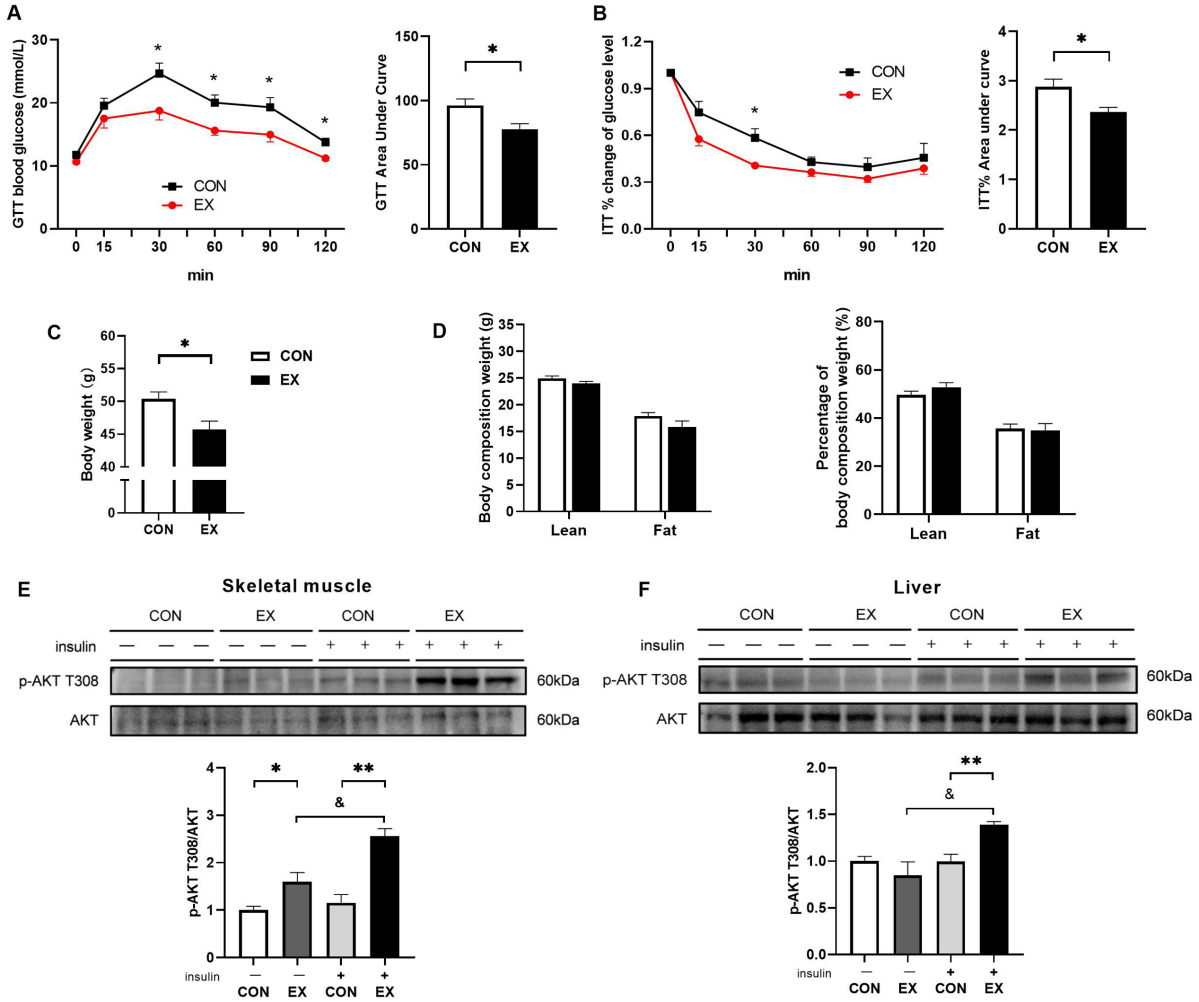
Effect of 12-week aerobic exercise on glucose tolerance test **(A)**, insulin tolerance test result **(B)**, body weight **(C)**, body composition **(D)**, and phosphorylation of AKT T308 in skeletal muscle **(E)** and liver **(F)**. *p < 0.05, **p < 0.01, &*p* < 0.05.

As expected, aerobic exercise attenuated IR in DIO mice. As shown in Figure 3, 12-week aerobic exercise significantly improved glucose tolerance (Figure 3A) and insulin tolerance (Figure 3B). While exercise effectively reduced body weight (Figure 3C), it did not induce significant alterations in the body composition of the mice (Figure 3D). Notably, Exercise significantly enhanced the insulin-induced phosphorylation of Protein Kinase B (AKT) at T308 in skeletal muscle (Figure 3E) and liver (Figure 3F).

### 3.3 BCAAs supplementation increased plasma BCAAs and BCKAs levels

**Figure 4:**
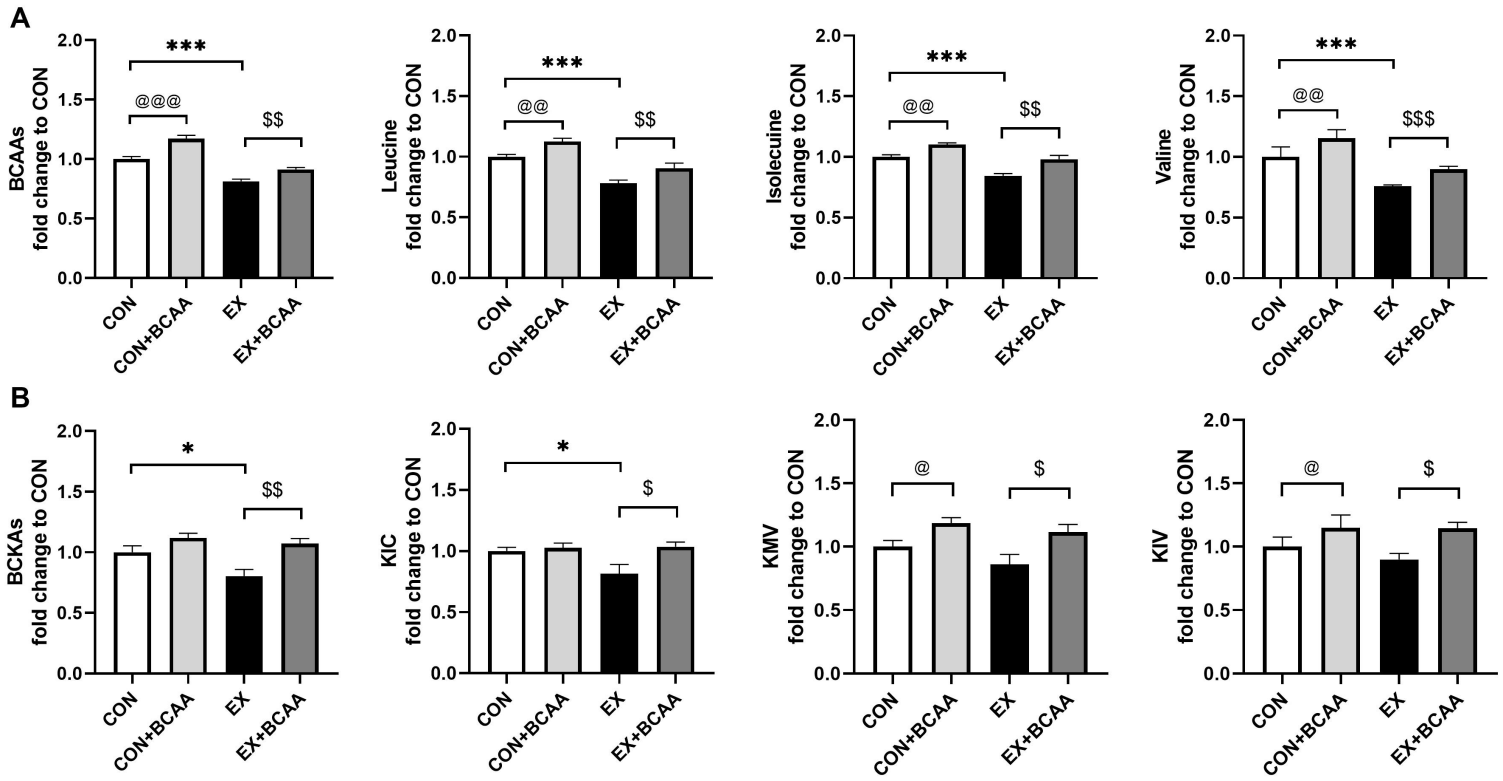
Effect of exercise intervention and BCAAs supplementation on plasma BCAAs **(A)** and BCKAs **(B)** (relative to CON group). *p < 0.05, ***p < 0.001, @*p* < 0.05, @@*p* < 0.01, @@@*p* < 0.001, $*p* < 0.05, $$*p* < 0.01, $$$*p* < 0.001. BCKA, branched-chain keto acids; KIC, α-ketoisocaproic acid; KMV, α-keto-β-methylvaleric acid; KIV, α-ketoisovaleric acid.

We then explored whether the lower BCAAs mediated exercise’s beneficial effect on IR. The DIO mice were subjected to exercise with or without BCAAs in drinking water. 12-week BCAAs supplementation significantly elevated the levels of plasma BCAAs (Figure 4A) and BCKAs (Figure 4B).

### 3.4 BCAAs supplementation attenuated the exercise-improved insulin sensitivity

**Figure 5:**
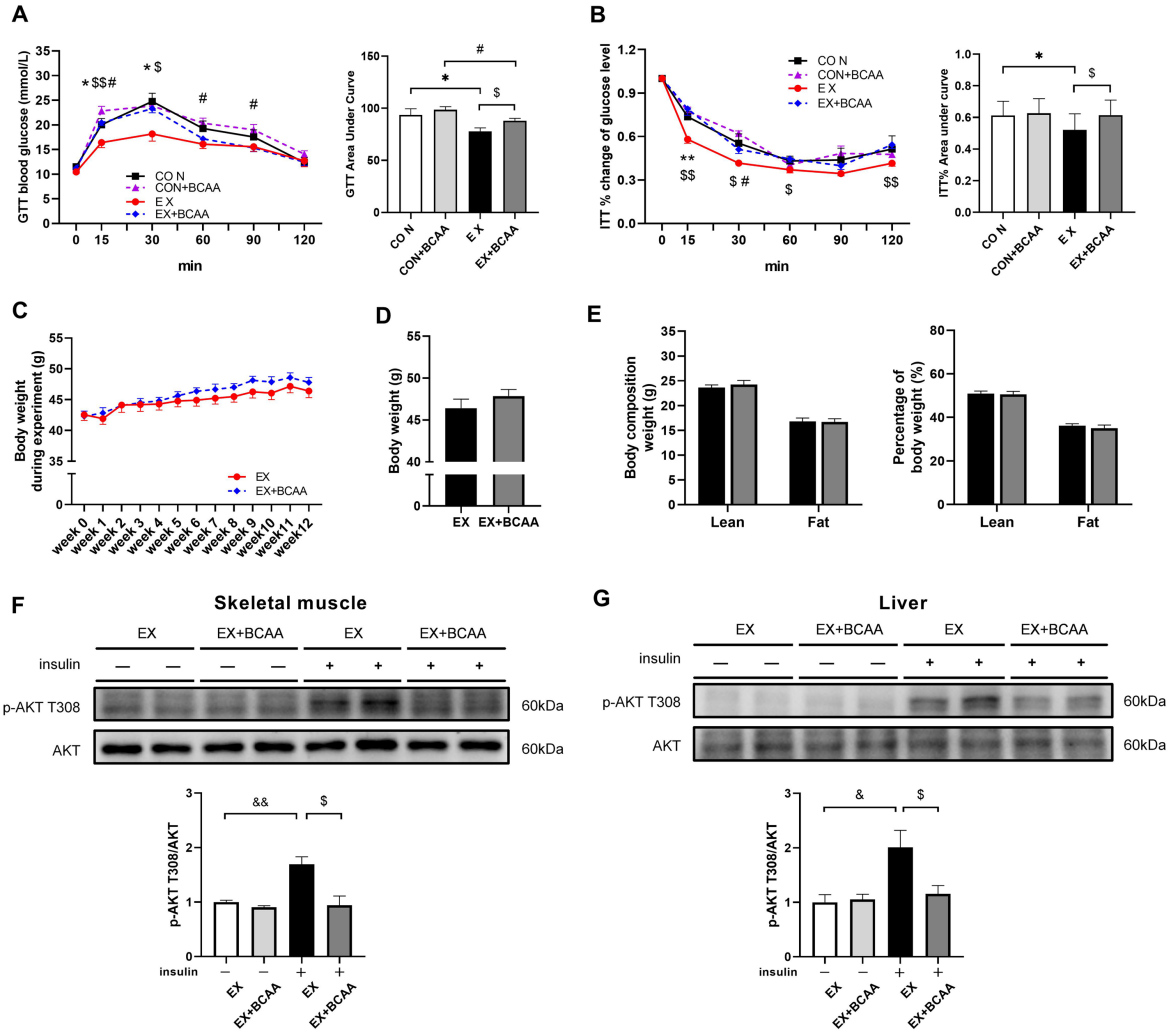
Effect of exercise intervention and BCAAs supplementation on glucose tolerance test **(A)**, insulin tolerance test result **(B)**, the change of body weight during 12-week of exercise **(C)**, body weight after 12-week of exercise **(D)** and body composition **(E)**, and phosphorylation of AKT T308 in skeletal muscle **(F)** and liver **(G)**. *p < 0.05, #*p* < 0.05, &*p* < 0.05, &&*p* < 0.01, $*p* < 0.05.

As shown in Figure 5, the exercise-induced improvements of glucose tolerance (Figure 5A) and insulin sensitivity (Figure 5B) were significantly attenuated after BCAAs supplementation. BCAAs supplementation did not result in significant difference in body weight (Figures 5C,D) or body composition in the exercise group (Figure 5E). Further, compared with that in the exercise group, AKT activity in skeletal muscle (Figure 5F) and liver (Figure 5G) was significantly decreased by BCAAs supplementation, indicating an impaired insulin signaling.

### 3.5 BCAAs supplementation did not affect mTORC1 activity in response to exercise intervention

**Figure 6:**
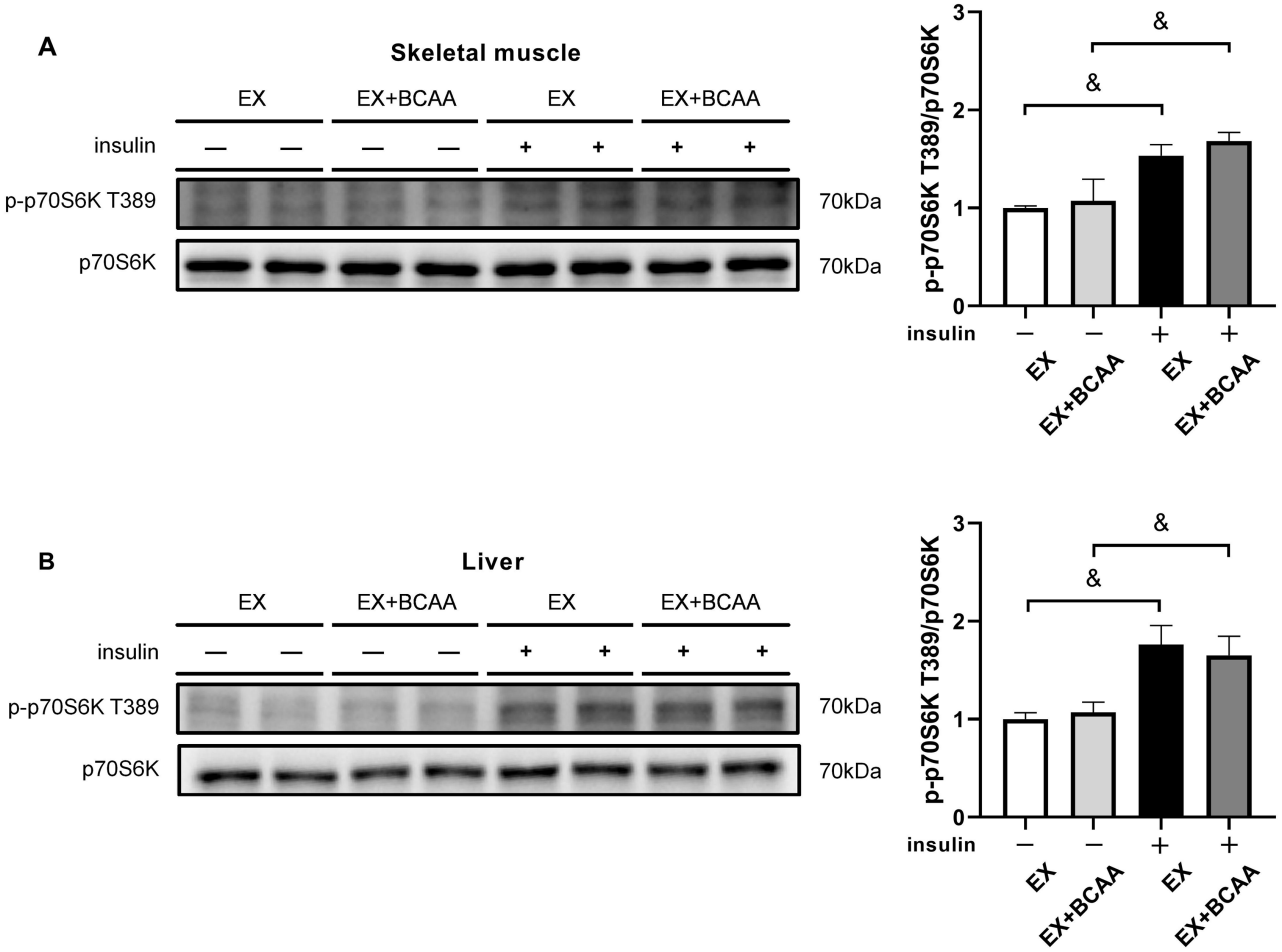
Effect of exercise intervention and BCAAs supplementation on the phosphorylation of p70S6K T389 in skeletal muscle **(A)** and liver **(B)**. &*p* < 0.05, &&*p* < 0.01.

The T389 of 70 kDa Ribosomal S6 Kinase (p70S6K) can be phosphorylated by mTORC1. Unexpectedly, p70S6K phosphorylation in skeletal muscle (Figure 6A) and liver (Figure 6B) was not enhanced by BCAAs supplementation with or without insulin stimulation. These results suggested the detrimental effect of BCAAs supplementation on insulin sensitivity was independent of mTORC1 activity.

### 3.6 Exercise-enhanced AMPK activity in skeletal muscle and liver was abolished by BCAAs supplementation

**Figure 7:**
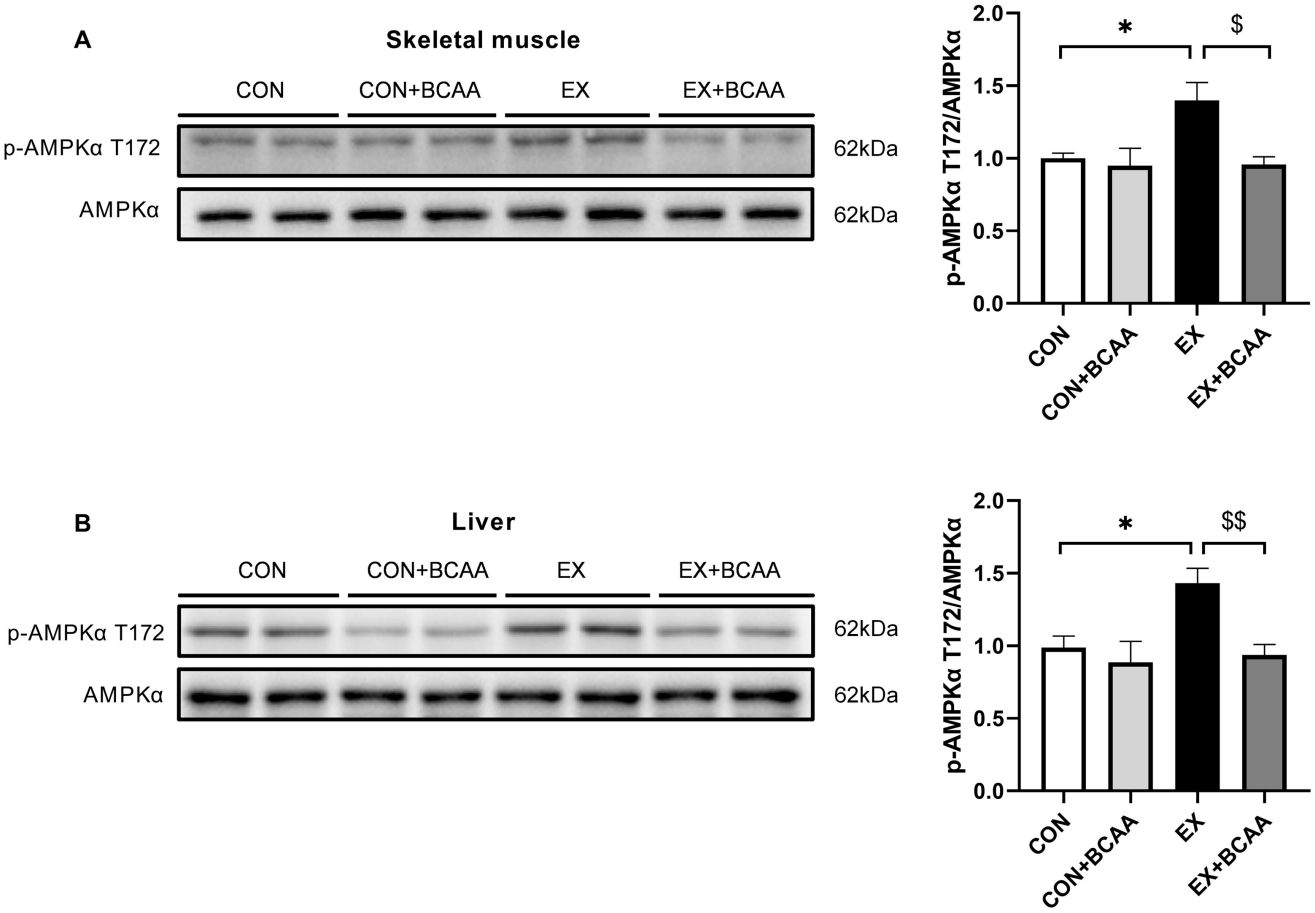
Effect of exercise intervention and BCAAs supplementation on the phosphorylation of AMPKα T172 in skeletal muscle **(A)** and liver **(B)**. **p < 0.01, $*p* < 0.05, $$*p* < 0.01.

The AMPK activity in tissues after exercise intervention and BCAAs supplementation was measured. Phosphorylation of the T172 residue in the activating loop of the *α*-subunit is the primary activator of AMPK. As expected, exercise increased the phosphorylation of AMPKα T172 in skeletal muscle (Figure 7A) and liver (Figure 7B). Importantly, BCAAs supplementation abolished this exercise-increased AMPK activity. These results indicated AMPK mediated the impacts of exercise-BCAAs axis on IR.

## 4 Discussion

In the current study, we found 12-week aerobic exercise reduced the plasma BCAAs levels and stimulated the expression of BCAAs catabolic enzymes in skeletal muscle and liver in obese mice, accompanied with attenuated IR. Importantly, BCAAs supplementation elevated plasma BCAAs and abolished the beneficial effects of exercise on insulin sensitivity, suggesting that the restoration of BCAAs homeostasis mediated the exercise-improved insulin sensitivity. AMPK activation likely played a role in the beneficial effects of exercise-BCAAs axis on insulin sensitivity.

Numerous studies of the effects of exercise on plasma BCAAs level have been performed in human (15, 21, 22, 25–29) and rodents (16, 30–32), yielding inconsistent results. While some studies suggest that exercise training can lower elevated BCAAs levels associated with metabolic disorders like obesity (26, 32), diabetes mellitus (25, 27, 30), and obesity with prediabetic status (31). Contrary findings, however, report no discernible effects of exercise on BCAAs (15, 16, 22, 28, 29). The inconsistence may be due to variations in exercise protocols and loads (33), as well as nutritional states of the subjects (21). Our data revealed that aerobic exercise training reduced the plasma BCAAs levels and attenuated IR in DIO mice, corresponding to increased expression of BCAAs catabolic enzymes in skeletal muscle and liver, thereby contributing to reduced BCAAs levels. These findings imply a potential causal link between BCAAs and the positive impact of exercise on IR. Notably, this hypothesis was further supported by our intervention, which offset the decline in BCAAs concentrations resulting from exercise training through targeted BCAAs supplementation. A recent investigation employing similar long-term aerobic exercise combined with BCAAs supplementation in DIO mice reported causal relationships akin to those identified in our present study (32). Together, these studies provided evidence and underlying mechanism for the beneficial effects of long-term aerobic exercise on the prevention and intervention of diabetes.

The impact of amino acid on IR under conditions of metabolic disorders is similarly complex. Organisms with metabolic abnormalities exhibit intricate responses to amino acid supplementation, especially in relation to IR. Prior investigations have linked the activation of mTORC1 and its downstream targets by excess BCAAs, most notably leucine, with increased IR. This connection is evident when obese rodents are supplemented with BCAAs, leading to the activation of mTORC1 and subsequent downstream effectors (10, 11). Moreover, treatment with rapamycin, a specific inhibitor of mTORC1, has been shown to partially mitigate the IR induced by BCAAs (10). Our data show BCAAs supplementation impairs insulin signaling without enhancing mTORC1 activity, excluding the involvement of mTORC1. This may involve exercise training enhancing the catabolism of BCAAs while augmenting lean mass (21), with BCAAs upregulating mTORC1, a primary pathway controlling protein translation and synthesis (34).

On the other hand, AMPK mediates numerous the benefits of exercise (35). AMPK is at the nexus of metabolic signaling pathways, and energy depletion is indicated by AMPK phosphorylation, which also can inhibit mTORC1 signaling (36). Our data indicate that exercise increased AMPK activity in skeletal muscle and liver as expected. Importantly, this increase was blocked by BCAAs supplementation. Similar studies by Saha et al. (36) and Coughlan et al. (37) have found through *in vitro* experiments that excess leucine can impair insulin signaling pathways by inhibiting AMPK activity in rat extensor digitorum longus cells. Thus, it is likely that AMPK mediates the beneficial effects of exercise-BCAAs axis. Meanwhile, BCAAs have been reported to activate AMPKα2 in adipocytes and stimulate lipolysis, leading to hepatic fatty acid accumulation (38). Chronic accumulation of fatty acids in heart induced by BCAAs appears to contribute to myocardial injury (39). The underlying mechanism remains to be investigated.

## 5 Conclusion

In summary, the present study shows that long-term aerobic exercise reduces the circulating BCAAs to improve insulin sensitivity in DIO mice, unraveling a mechanism underlying the beneficial effects of exercise. In addition, the influences of dietary BCAAs intake on exercise-attenuated IR suggest that nutritional protein intake may affect the therapeutic effects of exercise, highlighting the combined exercise and nutrition intervention strategy for diabetes management. Exercise boosted AMPK activity, inhibited by BCAAs supplementation, implying the role of AMPK in the exercise-BCAAs interplay on IR.

## Data Availability

The raw data supporting the conclusions of this article will be made available by the authors, without undue reservation.
